# Supplementary material for: Low vs standardized dose anticoagulation regimens for extracorporeal membrane oxygenation: A meta-analysis
Source: PLoS One. 2021 Apr 8;16(4):e0249854. doi: 10.1371/journal.pone.0249854 (PMC8031334; doi:10.1371/journal.pone.0249854)
Supplement: Attachment — Submitted filename: Response to Reviewers.docx [file pone.0249854.s002.docx]

Dear Editors and Reviewers:

Thank you for your letter and for the reviewers’ comments concerning our manuscript entitled “Low vs standardized dose anticoagulation regimens for extracorporeal membrane oxygenation: A Meta-analysis” (PONE-D-20-35042). Those comments are all valuable and very helpful for revising and improving our paper. We have studied comments carefully and the responds to the reviewer’s comments are as following:

1. Is the manuscript technically sound, and do the data support the conclusions?

The manuscript must describe a technically sound piece of scientific research with data that supports the conclusions. Experiments must have been conducted rigorously, with appropriate controls, replication, and sample sizes. The conclusions must be drawn appropriately based on the data presented.

Reviewer #1: No

The author replied：I would like to thank the reviewers for their valuable advice. The reviewer replied to no because we included letter in the study. First of all, I would like to thank the reviewers for their review of our manuscript. Meta analysis, as a secondary literature study, may be comprehensive, and whether it can be included depends on the quality of the included study. The letter included in this study meets the inclusion criteria, and it reports the grouping methods, research results and so on. We conducted a bias risk analysis and concluded that it met our inclusion criteria. Please fully consider our point of view, if there are still problems, we can change and re-analyze.

2. Has the statistical analysis been performed appropriately and rigorously?

Reviewer #1: No

The author replied：I would like to thank the reviewers for their valuable advice.The reviewer replied to no because we included letter in the study.

First of all, I would like to thank the reviewers for their review of our manuscript. Meta analysis, as a secondary literature study, may be comprehensive, and whether it can be included depends on the quality of the included study. The letter included in this study meets the inclusion criteria, and it reports the grouping methods, research results and so on. We conducted a bias risk analysis and concluded that it met our inclusion criteria. Please fully consider our point of view, if there are still problems, we can change and re-analyze.

3. Have the authors made all data underlying the findings in their manuscript fully available?

The PLOS Data policy requires authors to make all data underlying the findings described in their manuscript fully available without restriction, with rare exception (please refer to the Data Availability Statement in the manuscript PDF file). The data should be provided as part of the manuscript or its supporting information, or deposited to a public repository. For example, in addition to summary statistics, the data points behind means, medians and variance measures should be available. If there are restrictions on publicly sharing data—e.g. participant privacy or use of data from a third party—those must be specified.

Reviewer #1: No

The author replied：First of all, I would like to thank the reviewers for their review of our manuscript. Meta analysis as a secondary literature study, we have provided all the data that can support our conclusions, and our research is based on published literature. I wonder if there are any other requests. We look forward to hearing from you very much.

4. Is the manuscript presented in an intelligible fashion and written in standard English?

PLOS ONE does not copyedit accepted manuscripts, so the language in submitted articles must be clear, correct, and unambiguous. Any typographical or grammatical errors should be corrected at revision, so please note any specific errors here.

Reviewer #1: Yes

The author replied：Thanks

5. Review Comments to the Author

Please use the space provided to explain your answers to the questions above. You may also include additional comments for the author, including concerns about dual publication, research ethics, or publication ethics. (Please upload your review as an attachment if it exceeds 20,000 characters)

Reviewer #1: I have two main concerns:

1. Authors present clear criteria for standard doses of heparin (APTT 50-70 sec, ACT 180-220 sec). However there are no criteria for low-dose heparin strategy. What level of APTT, or/and ACT reflect low-dose strategy? If there are no clear criteria, how can authors do any conclusions in comparison of standard vs low doses of heparin during ECMO?

2. For analysis authors use posters and even letters. I’m not sure, that these forms of publications are worthy for meta-analysis.

The author replied to # 1：You have a very good point. In this meta analysis，patients were included in the standard doses of heparin group if they were started on a continuous infusion of anticoagulant after initiation of ECMO, which was monitored with ACT with a target of 180–220 s or APTT target of 50–70 s. The low-heparin group was supported with an low-dose strategy during ECMO, which included 5000 units of heparin intravenously at the time of ECMO initiation and ongoing, received an low-dose protocol (ACT goal: 140–160 s or APTT < 45 s), or did not receive systemic anticoagulation. Since heparin was not used continuously in the follow-up, the bleeding measurements such as ACT or APTT were not continuous monitored. However, compared with the standard dose, it also belongs to low-dose anticoagulation. The purpose of this study was to compare the advantages and disadvantages of low and standardized dose anticoagulation regimens, so it was also included in this study. Please fully consider our point of view.

The author replied to # 2：First of all, I would like to thank the reviewers for their review of our manuscript. Meta analysis, as a secondary literature study, may be comprehensive, and whether it can be included depends on the quality of the included study. The letter included in this study meets the inclusion criteria, and it reports the grouping methods, research results and so on. We conducted a bias risk analysis and concluded that it met our inclusion criteria. Please fully consider our point of view, if there are still problems, we can change and re-analyze.
